# Synthesis of poly(vinyl alcohol) by blue light bismuth oxide photocatalysed RAFT. Evaluation of the impact of freeze/thaw cycling on ice recrystallisation inhibition†

**DOI:** 10.1039/d2py00852a

**Published:** 2022-07-29

**Authors:** Ioanna Kontopoulou, Thomas R. Congdon, Simon Bassett, Ben Mair, Matthew I. Gibson

**Affiliations:** Department of Chemistry, University of Warwick Coventry CV4 7AL UK m.i.gibson@warwick.ac.uk +44 (0)247 652 4112; Division of Biomedical Sciences, Warwick Medical School, University of Warwick Coventry CV4 7AL UK; Cryologyx Ltd 71-75 Shelton Street London WC2H 9JQ UK; Synthomer (UK) Ltd Central Road Templefields Harlow Essex CM20 2BH UK

## Abstract

Poly(vinyl alcohol), PVA, is the most potent polymeric ice recrystallisation inhibitor (IRI), mimicking a complex function of ice binding proteins. The IRI activity of PVA scales with its molecular weight and hence broad molecular weight distributions in free radical-derived PVAs lead to activity measurements dominated by small amounts of heavier fractions. Well-defined PVA can be prepared by thermally initiated RAFT/MADIX polymerization using xanthates by the polymerization of the less activated monomer vinyl acetate. The low conversions and molecular weights obtained during this approach, often requires feeding of additional initiator and bulk polymerization. Here we employ bismuth oxide photo-RAFT in solution, using blue light (450 nm), rather than previously reported white light, to obtain a library of PVA's. The use of blue light enabled quantitative conversion and acceptable dispersities. Purple light (380 nm) was also used, but asymmetric molecular weight distributions were obtained in some cases. High concentrations of high molecular weight PVA is known to form cryogels during freeze/thaw which has led to speculation this might limit the use of PVA in environments where the temperature cycles *e.g.* the construction industry. After 4 freeze/thaw cycles there was only small changes in observable IRI for all synthesised PVAs and two commercial standards. In an extended test, activity was retained after 100 freeze/thaw cycles, mitigating concerns that PVA could not be used in situations where freeze/thaw cycles occur. This work presents a convenient method to obtain well-defined PVAs for cryoscience studies compared to conventional thermal-RAFT and indicates that cryogelation concerns may not prevent their use.

## Abstract 2

Poly(vinyl alcohol) is synthesised using blue light bismuth oxide photo-RAFT, to high conversion. The ice recrystallisation inhibition activity of these polymers was shown to be retained over 100 freeze/thaw cycles.

## Graphical abstract

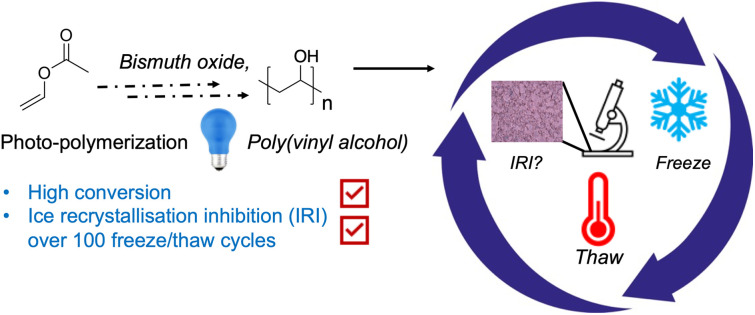

## Introduction

Nature has evolved specialised macromolecules which are capable of binding to and modulating ice formation and growth, including ice binding proteins^1^ and also polysaccharides.^2,3^ Along with the basic science of how these ice binding proteins (or other macromolecules) can recognise ice in a large excess of water, they have been explored for low temperature applications such as the cryopreservation of cells^4,5^ or to modulate ice growth in frozen food.^6^ For many translational applications synthetic mimics of proteins are desirable, but must be scalable, durable and tunable.^7,8^ Small molecule ice growth inhibitors have been reported^9–12^ as have self-assembled materials,^13,14^ bespoke peptides^15,16^ and graphenics.^17,18^ One of the most active synthetic mimics reported to date is poly(vinyl alcohol) (PVA).^19–21^ PVA can bind the prismatic faces of growing ice crystals slowing the rate of ice recrystallisation^20,22,23^ at concentrations below 1 mg mL^−1^. PVA is appealing for many applications as it is produced on multi-tonne scales, has low toxicity and is approved for use in food.^24^

Previous investigations of PVA (and other IRI's^7,8,25,26^) have focussed on biomedical^27,28^ or food^29,30^ applications but there are other technological areas where ice growth or formation is a challenge such as wind turbine blades^31^ or plane wings.^32^ When concrete is exposed to freeze/thaw cycles, ice growth compromises its function, reducing the useful life span of the material. Srubar *et al.* have shown that PVA-based comb-polymers can limit ice growth at the high pHs of concrete (>12) reducing the freeze–thaw associated damage.^33^ The authors noted that branched PVA was required to avoid irreversible aggregation of linear PVA, which may reduce the IRI activity over a large number of freeze–thaw cycles. However, there is limited evidence on freeze/thawing on the IRI activity of linear PVA. Poly(vinyl alcohol) is known to form cryogels, which are typically prepared by freeze/thaw of concentrated solutions at ∼50 mg mL^−1^ of high molecular weight PVA.^34–36^ However, this is 50-fold more concentrated than what is required for IRI. There is, to the best of our knowledge, no detailed study on the actual impact of freeze/thaw cycling of PVA solutions on their IRI activity and no consideration of the impact of molecular weight.

Commercial PVA is polydisperse and often is a copolymer with residual acetate groups, derived from the precursor poly(vinyl acetate) and hence studies on PVA's IRI require precision materials.^21,37^ Unlike activated monomers, such as (meth)acrylates, vinyl esters are more challenging to polymerize by controlled radical polymerization, necessitating the use of xanthates in RAFT/MADIX polymerization.^38,39^ Müllner and co-workers have demonstrated that vinyl acetate can be polymerized under white light irradiation by addition of a bismuth oxide photocatalyst^40^ offering a more convenient synthetic route than traditional thermal initiation. Photo-polymerization offers unique opportunities for macromolecular design and spatio-temporal control of polymerization.^41–46^

Here we report the synthesis of well-defined poly(vinyl alcohol) by blue-light mediated bismuth oxide photocatalysed polymerization. This method is shown to produce high conversions (>90%) and narrow dispersities. Following removal of the acetate groups, the panel of polymers was evaluated for IRI activity, showing that after a single freeze/thaw cycle all molecular weight fractions retained activity. A smaller set of polymers were then exposed to up to 100 freeze/thaw cycles and in all cases IRI activity was retained. This work shows that at a concentration range relevant for IRI activity, PVA does not lose function – mitigating concerns about its durability for extended applications, such as in construction.

## Results and discussion

**Fig. 1:**
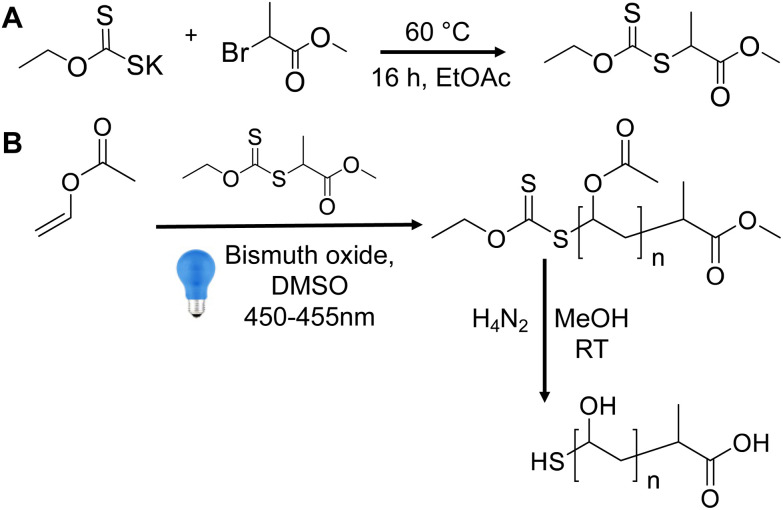
(A) Synthesis of 2-(ethoxycarbonothioyl)sulfanyl propanoate (EXEP – MADIX/RAFT agent); (B) synthesis of poly(vinyl acetate) and subsequent deprotection using hydrazine to give poly(vinyl alcohol).

Thermally initiated RAFT/MADIX polymerization of vinyl acetate often fails to proceed to full conversion; to obtain higher molecular weight polymers and higher conversion requires the feeding of more initiator over time.^21,39^ To overcome this, a bismuth oxide photocatalyst was employed, as first reported by Müllner and co-workers.^40^ The RAFT/MADIX agent, 2-(ethoxycarbonothioyl)sulfanyl propanoate (EXEP) was synthesised^21^ (Fig. 1A) and used to polymerize vinyl acetate (Fig. 1B).

**Fig. 2:**
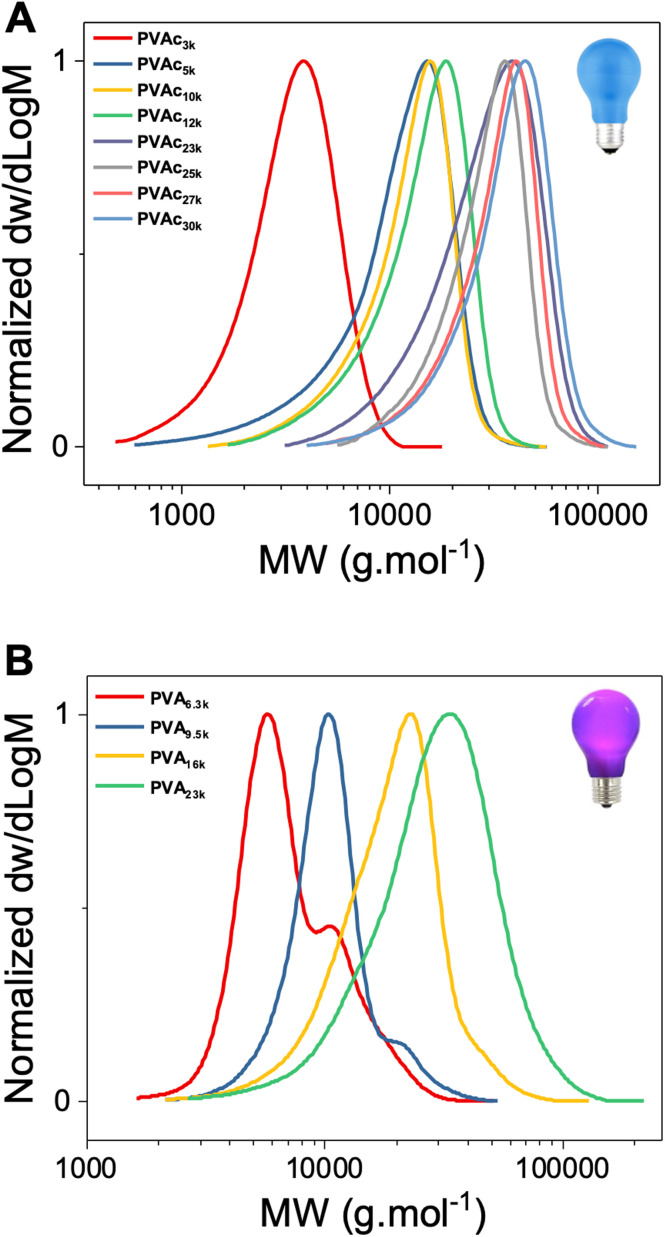
Molecular weight distributions of poly(vinyl acetates) from size exclusion chromatography using THF as the eluent. (A) Polymers prepared using blue light (450 nm); (B) polymers prepared using purple light (380 nm).

**Fig. 3:**
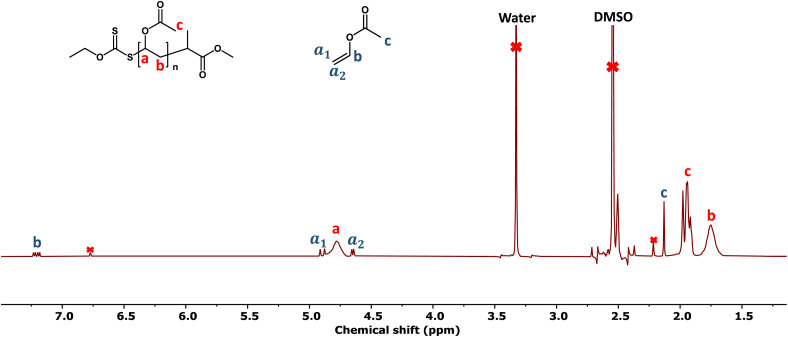
Example ^1^H NMR spectrum of a PVAc obtained by blue light bismuth oxide photopolymerization, collected in DMSO-*d*_6_ conversion = 91%.

In the report by Müllner and co-workers,^40^ white light from a household lightbulb was used, which led to vinyl acetate conversions of ∼70%. One of our aims was to achieve higher conversions to reduce wastage of monomer and facilitate purification, so we explored using more focussed light sources. Blue (450 nm, 18 W) and purple (380 nm, 18 W) light sources were explored using a range of monomer to CTA ratios. Polymers were obtained in all cases, but the purple light polymerizations resulted in bimodal distributions or “shoulders”, especially at lower feed ratios (Fig. 2B). The purple light is absorbed by the CTA, so it is feasible that direct photolysis of the RAFT agent can occur, as has been reported previously for VAc polymerization (with 365 nm irradiation).^47^ In contrast the blue light has less overlap with the CTA's absorption it will not photolyze the CTA efficiently and hence proceed primarily *via* activation of the bismuth oxide. Direct blue light polymerization of VAc using MADIX agents is possible, but less than 50% conversion is achieved in 24 hours,^48^ highlighting the benefit of the bismuth oxide photocatalyst. Fig. 3 shows an example NMR spectra from polymerization of VAc with bismuth oxide highlighting the high conversion (>90%) of monomer to polymer, in contrast to our experience of thermally initiated vinyl acetate polymerizations where lower conversion is typical. Fig. 3 shows example SEC traces from a range of polymerizations using blue and purple light targeting different degrees of polymerization (by varying the [monomer] : [CTA] ratio), showing monomodal distributions for blue light, but broader distributions for purple with “shoulders” in some cases.

This blue-light method ensured predictable molecular weights and acceptable dispersities were obtained, as summarised in Table 1. Conversion was determined in each case by ^1^H NMR, by comparing the residual monomer peak to the polymer backbone. The smallest PVAs (needed for the IRI testing) were obtained by targeting DP 100, but deliberately stopping the reaction (removing from light) at lower conversion. In all other cases, high conversions above 90% were achieved. A control reaction with no CTA present led to no conversion. As with any photoreaction the power of the light source and any heat generated is important to consider; here the photoreactor was cooled by an internal fan (see ESI† for specifications).

**Table 1:** Poly(vinyl acetate) synthesised by blue-light bismuth oxide photocatalysis

PVAc	[M] : [CTA] : [Bi_2_O_3_]	Conv._NMR_ (%)	*M*_n,Theo_ (g mol^−1^)	*M*_nSEC_ (g mol^−1^)	*Đ*_SEC_	Irradiation time (hours)
PVAc_3k_	100 : 1 : 0.1	15	1290	2800	1.29	4
PVAc_8k_	100 : 1 : 0.1	66	5680	8000	1.53	5
PVAc_10k_	100 : 1 : 0.1	90	7750	10 600	1.14	11
PVAc_12k_	200 : 1 : 0.2	85	14 640	11 800	1.33	16
PVAc_23k_	300 : 1 : 0.3	91	23 500	23 000	1.41	20
PVAc_25k_	250 : 1 : 0.25	99	21 300	25 300	1.22	21
PVAc_27k_	300 : 1 : 0.3	98	25 300	27 200	1.24	20
PVAc_30k_	400 : 1 : 0.4	84	29 000	30 600	1.31	22
PVAc	100 : 0 : 0.1	0	0	0	0	16

**Fig. 4:**
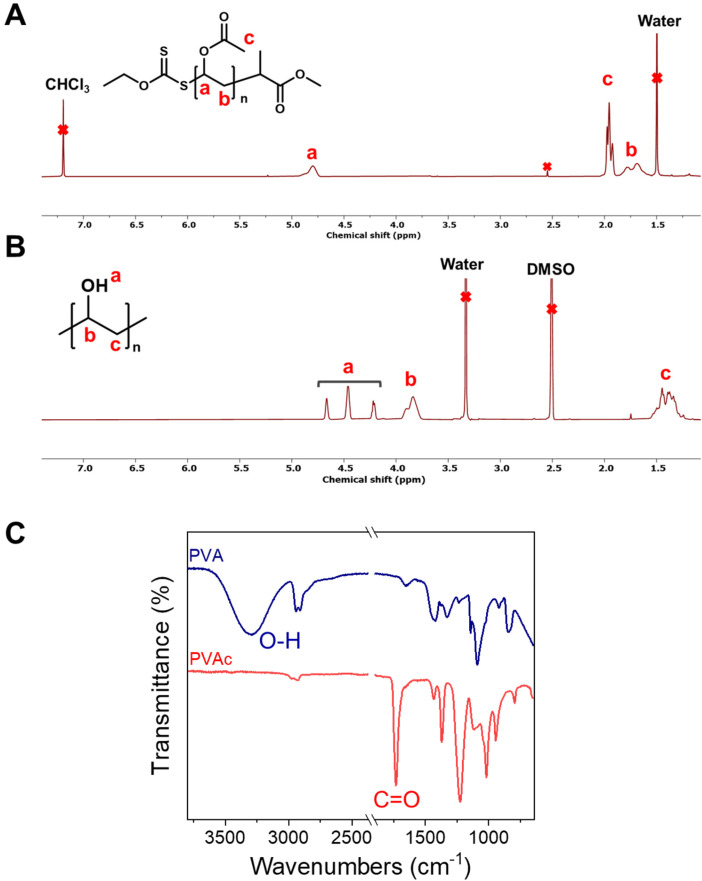
Characterization of the conversion of PVAc to PVA. (A) ^1^H NMR of PVAc in CDCl_3_; (B) ^1^H NMR of PVA in DMSO-*d*_6_; (C) infrared spectra of PVA and PVAc with key peaks indicated.

To obtain PVA, the acetate groups were removed from PVAc using hydrazine hydrate, which gives full conversion, unlike when *e.g.*, NaOH is used. ^1^H NMR confirmed quantitative removal of the acetate (Fig. 4A/B) and the resulting polymers were water soluble, unlike the PVAc precursor. Infrared spectroscopy confirmed complete removal of the acetate group (C=O) at 1730 cm^−1^ and introduction of O–H stretch at 3296 cm^−1^, Fig. 4C. All polymers were further purified by dialysis before further use to ensure no small molecule contaminants which would interfere with subsequent IRI activity assays.

**Fig. 5:**
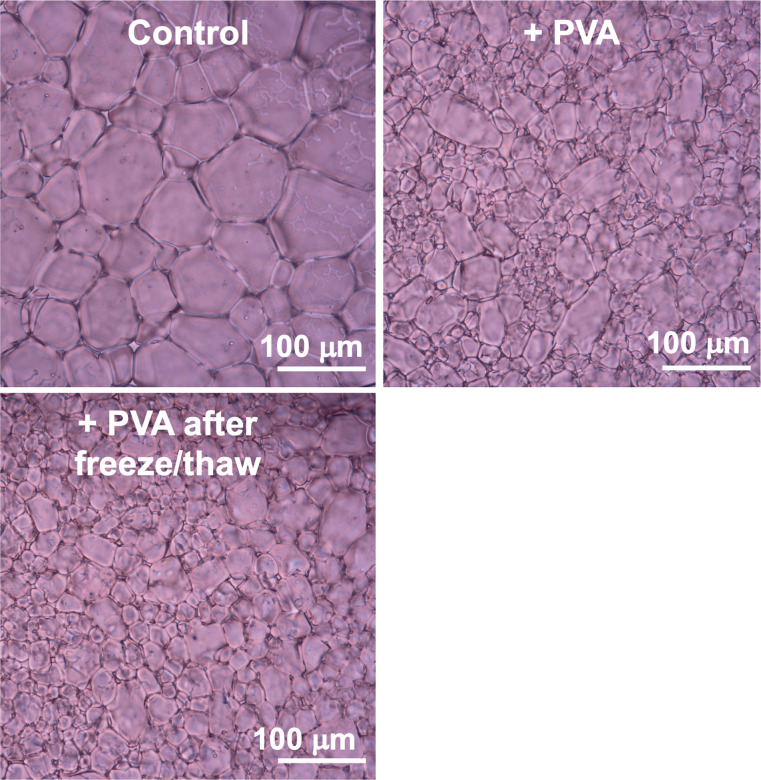
Example cryomicrographs of IRI assays. Control is PBS alone. [PVA_25k_] = 0.0625 mg mL^−1^. Concentration chosen where PVA begins to loose activity. Freeze (−20 °C)/thaw (ambient) cycle. Images are after 30 min annealing at −8 °C in PBS solution.

PVA and commercial PVA (which is technically PVA-*co*-PVAc as it not fully deacetylated) is well known to form cryogels when subjected to freeze/thaw. This typically requires higher molecular weight polymers and is conducted at concentrations above 5 wt%, which is approximately 50 mg mL^−1^,^35,36^ above the limiting concentration required for IRI activity.^20,21,49^ It has therefore been suggested that PVA might not be suitable in applications to control ice growth where repeated freeze/thaw cycles are used (unlike in cryopreservation where a solution is typically freeze/thawed once). Frazier *et al.* synthesised PEG-grafted PVA's and showed they can reduce freeze/thaw damage in concrete and hypothesised that linear PVA is not suitable due to cryogelation, but did not investigate freeze/thaw cycles of PVA.^33^ Therefore, we set out to investigate if PVA solutions in a concentration range relevant for IRI retain activity over multiple freeze/thaw cycles. To evaluate IRI activity the ‘splat’ assay was used.^21,49,50^ A 10 μL droplet of polymer in buffer is nucleated at −80 °C to produce small ice crystals, which are then annealed at −8 °C for 30 minutes. After this time the mean grain size (MGS) is determined by image analysis and the ice growth reported as a percentage of the buffer alone. Fig. 5 shows example micrographs of the buffer alone, with PVA and after a single freeze/thaw cycle of the PVA.

**Fig. 6:**
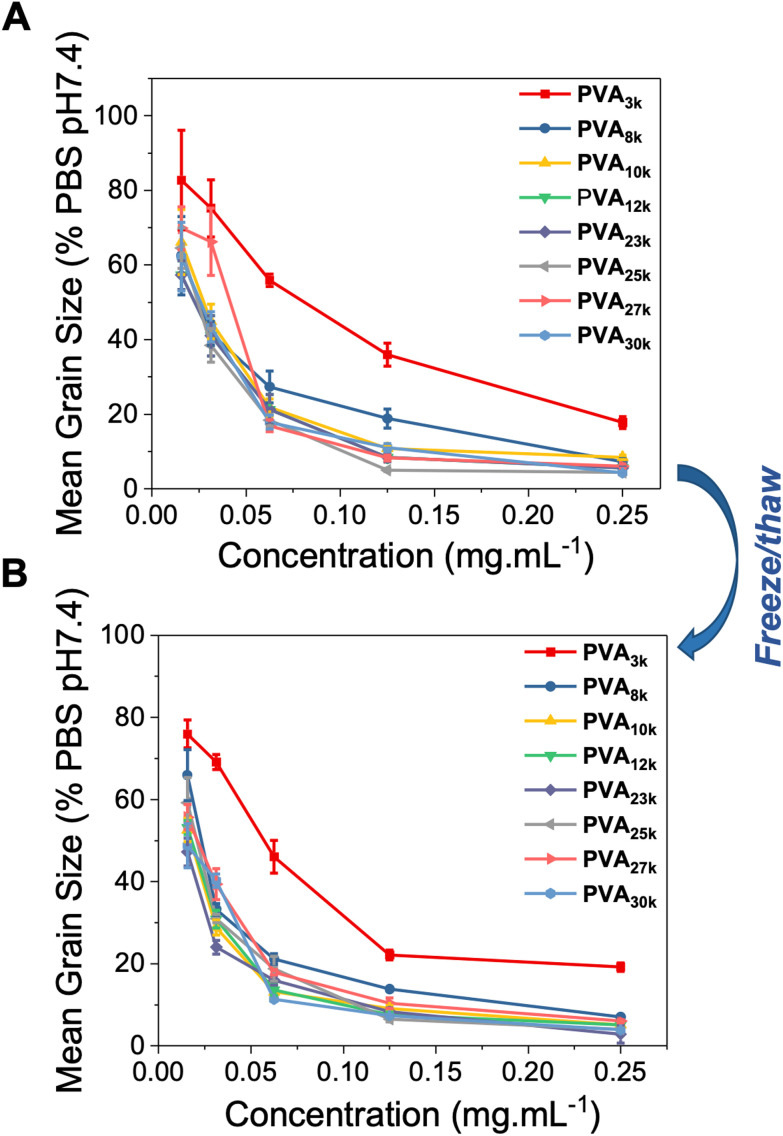
Ice recrystallisation inhibition activity of the PVA library before (A) and after (B) a freeze (−20 °C)/thaw (ambient) cycle. Assays were conducted in phosphate buffered saline, and the mean grain size determined after 30 minutes annealing at −8 °C.

Using the splat assay, as described above, the panel of PVAs was evaluated through multiple freeze/thaw cycles. A vial of each PVA at the indicated concentration was prepared, frozen (24 hours at −20 °C) and then thawed, before an aliquot was drawn and tested, and the remainder subjected to further freeze/thaw cycles. The results of this are shown in Fig. 6. The concentration range was chosen to ensure we analysed the range where PVA begins to lose activity, rather than too high where freeze/thaw induced aggregation might only deactivate a small fraction of PVA and lead to false-positive results. As can be seen, between Fig. 6A (before) and Fig. 6B (after freeze/thaw) there was minimal impact on the observable activity, showing it is tolerant to at least one freeze/thaw cycle.

**Fig. 7:**
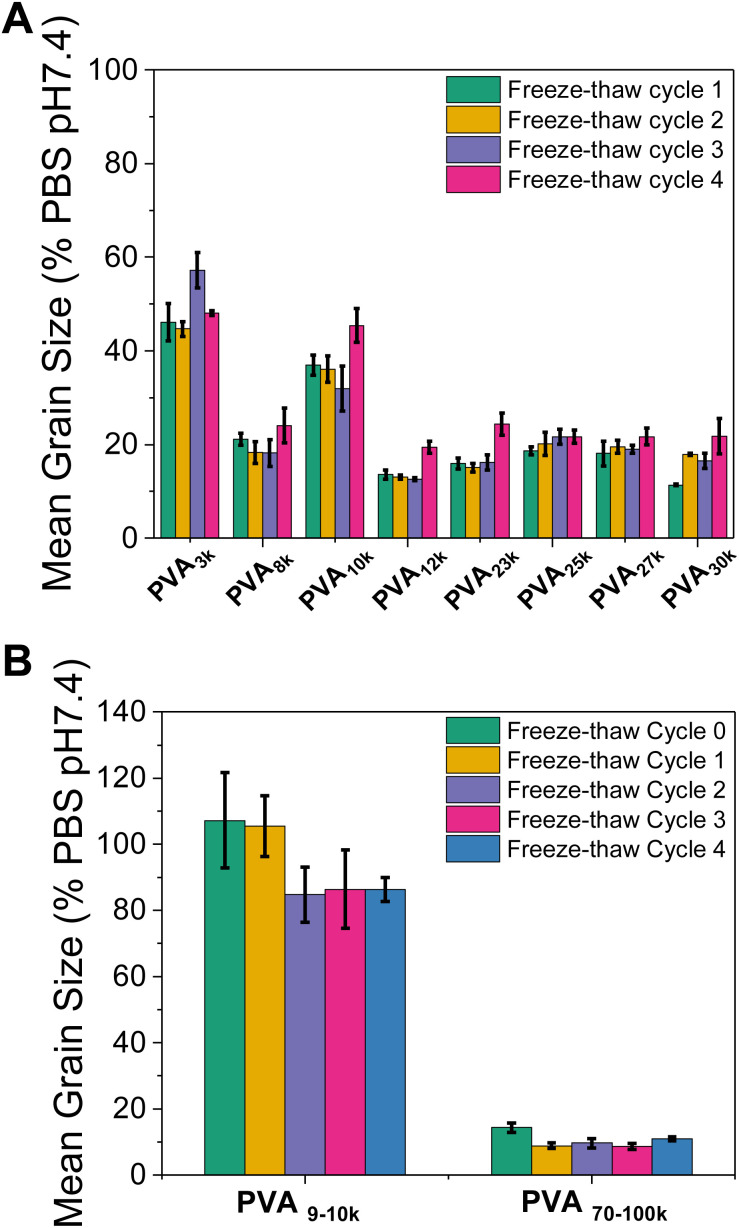
Ice recrystallisation inhibition activity of the PVA library over multiple freeze (−20 °C)/thaw (ambient) cycles. (A) PVAs from Table 1; (B) commercial PVA with manufacturer supplier *M*_w_ range (g mol^−1^). Assays were conducted in phosphate buffered saline, and the mean grain size determined after 30 minutes annealing at −8 °C.

Encouraged by the data above, a larger number of freeze/thaw cycles were undertaken, Fig. 7. These were undertaken at a fixed concentration of 0.0625 mg mL^−1^ where the PVAs begin to lose activity ensuring any deactivation effects are exaggerated and to ensure a larger number of cycles could be explored. This analysis was also undertaken using commercial PVA (Fig. 7B), which has higher dispersity values and is also partially acetylated (due to non-quantitative deacetylation). Across all the samples, there was a very small increase in MGS (*i.e.* reduction in activity) after 4 cycles. In general, IRI was retained even for the shortest PVAs (least active) and longest PVAs (which might be hypothesised to be more likely to irreversibly aggregate). This shows that across a moderate number of freeze/thaw cycles, solutions containing PVA retain their ability to prevent ice recrystallisation.

**Fig. 8:**
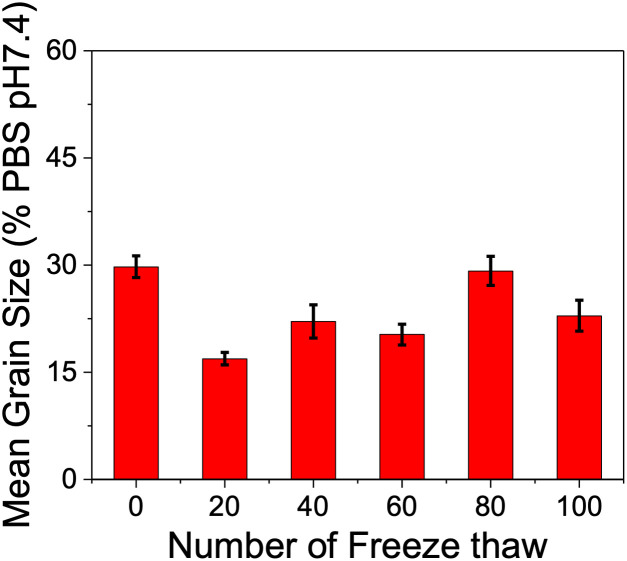
Ice recrystallisation inhibition activity of the PVA_12k_ at concentration of 0.0625 mg mL^−1^ after direct dissolution and after multiple freeze (liq. N_2_)/thaw (ambient) cycle. Assays were conducted in phosphate buffered saline, and the mean grain size determined after 30 minutes annealing at −8 °C.

As a final example, to provide a truly rigorous test of the freeze/thaw impact, which might be more representative of *e.g.* environmental application over a cold season, a single PVA (12k) was exposed to 100 freeze/thaw cycles with the IRI activity measured at 20 freeze/thaw intervals, Fig. 8. In this case, liquid nitrogen freezing was used, due to the practical challenge of 100 cycles. Over the course of the cycles, there was some variation in the absolute activity between 15 and 30% MGS, but with limited indication that more cycles led to loss of activity (Fig. S5†). It is important at this stage to note that aggregation which might deactivate the PVA, might also have the opposite effect of enhancing it. We have previously shown that adding in secondary, non-IRI active polymers to PVA can increase its activity due to depletion forces, forcing PVA onto the ice crystal.^51^ Molecular size is also correlated with the activity of several ice binding proteins, with larger proteins being more active.^52,53^ Aggregation has been proposed to enhance antifreeze glycoprotein activity, and aggregation (or assembly) of a type I antifreeze protein increases its activity.^52^ We have previous demonstrated that assembly of PVA, or other polymers, into nanoparticles can enhance activity in some cases.^54,55^ Hence, aggregation itself may occur, but in the dilute concentration range where PVA is active, it is clear that the macroscopic function of ice recrystallisation inhibition is retained and hence PVA can potentially be deployed in applications where multiple freeze/thaw cycles are expected.

## Conclusions

Here we have demonstrated that the photo-polymerization of vinyl acetate, using blue light irradiation and a bismuth oxide photocatalyst, provides well defined poly(vinyl acetates) with high conversion. This is an improvement compared to conventional thermal RAFT polymerizations which need re-feeding of initiator to drive conversion towards completion. Purple light was also shown to give rise to polymers but in some cases a “shoulder” in the molecular weight distributions was observed, which was attributed to a competing polymerization mechanism, where the xanthate was directly photolyzed. The library of poly(vinyl acetates) was converted to poly(vinyl alcohol) by quantitative deprotection using hydrazine. The library of PVAs was used to evaluate the impact of freeze/thaw cycles on the ice recrystallisation inhibition (IRI) activity of PVA. Specifically, the question of if irreversible aggregation due to cryogelation leads to deactivation of the PVA's IRI activity. A range of well-defined PVA's with degrees of polymerization from 10 to 250 were shown to retain all IRI activity following 4 freeze/thaw cycles, at concentrations relevant for IRI activity. Similarly, two commercial PVA's had a broadly similar profile. A single PVA was tested for 100 freeze/thaw cycles as a rigorous test of longevity of function, with no appreciable change in the observed IRI. This data shows that poly(vinyl alcohol) does not lose IRI activity during freeze/thaw stress and that easy to access linear PVA, rather than more complex branched architectures, can be used in applications where extensive freeze/thaw cycling occurs. The data presented here supports the exploration of PVA (and other IRI active materials) for application in transport or infrastructure where hundreds of freeze/thaw cycles are expected.

## Experimental section

### Materials

All chemicals were used as supplied. Ethyl acetate, hexane, methanol, dichloromethane, tetrahydrofuran, and magnesium sulphate were all purchased from Fisher Scientific at laboratory reagent grade. Deuterated chloroform (99.8 atom% D), dimethyl sulfoxide-d6 (99.9 atom% D), vinyl acetate (97%), potassium ethyl xanthate (96%), 2-(methyl bromopropionate) (98%), bismuth(iii) oxide powder (99.999% trace metals basis) hydrazine hydrate 50–60%, phosphate buffered saline tablet pH 7.4 (NaCl = 0.137 M and KCl = 0.0027 M at 25 °C), were purchased from Sigma Aldrich. Dialysis membrane (MWCO = 1000 Da/MWCO: 300–500 Da) was purchased from Spectra/Por.

### Physical and analytical methods

#### NMR spectroscopy

^1^H-NMR and ^13^C-NMR spectra were recorded at 300 MHz or 400 MHz on a Bruker DPX – 300 or DPX – 400 spectrometers respectively, with chloroform-*d* (CDCl_3_) and DMSO-*d*_6_ ((CD_3_)_2_SO) as the solvent. Chemical shifts of protons are reported as *δ* in parts per million (ppm) and are relative to tetramethylsilane (TMS) at *δ* = 0 ppm when using CDCl_3_ or solvent residual peak (CH_3_OH, *δ* = 3.31 ppm/DMSO, *δ* = 2.50 ppm).

#### Size exclusion chromatography

Size exclusion chromatography (SEC) analysis was performed on an Agilent Infinity II MDS instrument equipped with differential refractive index (DRI), viscometry (VS), dual angle light scatter (LS) and variable wavelength UV detectors. The system was equipped with 2× PLgel Mixed D columns (300 × 7.5 mm) and a PLgel 5 μm guard column. The mobile phase used was DMF (HPLC grade) containing 5 mM NH_4_BF_4_ at 50 °C at flow rate of 1.0 ml min^−1^. Poly(methyl methacrylate) (PMMA) standards (Agilent EasyVials) were used for calibration between 955 000–550 g moL^−1^. Analyte samples were filtered through a nylon membrane with 0.22 μm pore size before injection. Number average molecular weights (*M*_n_), weight average molecular weights (*M*_w_) and dispersities (*Đ*_M_ = *M*_w_/*M*_n_) were determined by conventional calibration and universal calibration using Agilent GPC/SEC software.

### Photo-polymerisation of vinyl acetate using bismuth oxide

As a representative example, into a 20 mL glass vial were added vinyl acetate (VA) (2.48 g, 28 mmoL, 300 eq.), 2-(ethoxycarbonothioyl)sulfanyl propanoate (EXEP) (0.02 g, 0.096 mmoL, 1 eq.), DMSO 2.60 mL and bismuth oxide (Bi_2_O_3_) (0.013 g, 0.028 mmol, 0.3 eq.). The vial was sealed with a subaseal. The sealed vial was incubated at 37 °C with magnetic stirring under 450–455 nm using the blue LED source in the photo-reactor for the specified times. An aliquot of crude polymerisation mixture was taken for ^1^H NMR conversion and *M*_n,NMR_ analysis in DMSO-*d*_6_. The sample was diluted in THF (15 mL) and centrifuged 3 times at RCF 22.769*g* remove Bi_2_O_3_ and subsequently precipitated into water to remove the DMSO. The polymer as then precipitated from THF into hexane (2 × 50 mL) to remove excess VAc and the polymer dried under high vacuum. Conversion (NMR): 98%, *M*_n_ (theoretical): 25 300 g moL^−1^. ^1^H NMR (CDCl_3_): *δ* = 1.55–1.89 (**C*H***_**2**_CHOOCH_3_, br, 2H), 1.88–2.01 (CH_2_CHOO**C*H***_**3**_, br, 3H), 4.74–4.93 (CH_2_**C*H***OOCH_3_, br, 1H). SEC (THF) *M*_nSEC_: 27 200 g mol^−1^, *Đ*_M_ = 1.24.

### Synthesis of poly(vinyl alcohol)

To obtain the poly(vinyl acetate) (PVA) it is necessary to hydrolyse the PVAc to remove the acetate protecting group. Into a 50 mL round bottom flask equipped with a stir bar, poly(vinyl acetate) (1.00 g) was dissolved in methanol (10 mL) and left to stir until dissolved. Hydrazine hydrate solution 50–60% (25 mL) was added and the mixture stirred under ambient conditions overnight. Afterwards, the methanol evaporate *in vacuo* and 50 mL milliQ water added in the round bottom flask and the polymer mixture dialysed (MWCO = 1000 Da) to remove unreacted hydrazine hydrate and methanol. The purified PVA was then lyophilised to give a white solid as the final product. ^1^H NMR (400 MHz, DMSO-d_6_): *δ* = 1.21–1.56 (**C*H***_**2**_CHOH, br, 2H), 3.75–3.94 (CH_2_**C*H***OH, br, 1H), (CH_2_CH**O*H***, br, 1H), 4.18–4.70.

## Conflicts of interest

There are no conflicts to declare.

## Supplementary Material

PY-013-D2PY00852A-s001
