# Supplementary material for: Synthesis of poly(vinyl alcohol) by blue light bismuth oxide photocatalysed RAFT. Evaluation of the impact of freeze/thaw cycling on ice recrystallisation inhibition
Source: Polym Chem. 2022 Jul 29;13(32):4692–700. doi: 10.1039/d2py00852a (PMC9379775; doi:10.1039/d2py00852a)
Supplement: PY-013-D2PY00852A-s001 [file PY-013-D2PY00852A-s001.pdf]

Electronic Supplementary Material (ESI) for Polymer Chemistry.
This journal is © The Royal Society of Chemistry 2022

Electronic Supplementary Information

Synthesis of Poly(vinyl alcohol) by Blue Light Bismuth Oxide Photocatalysed RAFT. Evaluation of the Impact of Freeze/Thaw Cycling on Ice Recrystallisation Inhibition

Ioanna Kontopoulou,[a] Thomas R Congdon,[b,c] Simon Bassett,[d] Ben Mair,[d] Matthew I. Gibson[a,b,*]

[a)] Department of Chemistry, University of Warwick, Coventry, CV4 7AL, United Kingdom

[b)] Division of Biomedical Sciences, Warwick Medical School, University of Warwick, Coventry, CV4 7AL, United Kingdom

[c)] Cryologyx Ltd, 71-75 Shelton Street, London, UK, WC2H 9JQ

[d)] Synthomer (UK) Ltd, Central Road, Templefields, Harlow, Essex, CM20 2BH, UK

CORRESPONDING AUTHOR DETAILS

*Fax: +44 247 652 4112. E-mail: m.i.gibson@warwick.ac.uk

Contents

1. **Experimental Section**......2
1.1. **Materials and Methods**......2
1.2. **Materials**......2
1.3. **Characterisation Techniques**......2
1.4. **Experimental Methods**......3
2. **Synthetic Methods**......5
3. **Supplementary Characterisation Data**......6
4. **References**......9

1. Experimental Section

1.1. Materials and Methods

1.2. Materials

All chemicals were used as supplied. Ethyl acetate, hexane, methanol, dichloromethane, tetrahydrofuran, and magnesium sulphate were all purchased from Fisher Scientific at laboratory reagent grade. Deuterated chloroform (99.8 atom %D), dimethyl sulfoxide-d6 (99.9 atom %D), vinyl acetate (97.0 %), potassium ethyl xanthate (96 %), 2-(methyl bromopropionate) (98 %), bismuth (III) oxide powder (99.999 % trace metals basis) hydrazine hydrate 50 – 60 %, phosphate buffered saline tablet pH 7.4 (NaCl = 0.137 M and KCl = 0.0027 M at 25 °C), were purchased from Sigma Aldrich. Dialysis membrane (MWCO = 1000Da/ MWCO: 300 – 500D) was purchased from Spectra/Por.

1.3. Characterisation Techniques

NMR Spectroscopy.

^{1}H-NMR and ^{13}C-NMR spectra were recorded at 300MHz or 400MHz on a Bruker DPX – 300 or DPX – 400 spectrometers respectively, with chloroform-*d* ($CDCl_3$) and DMSO-d_6 ($(CD_3)_2SO$) as the solvent. Chemical shifts of protons are reported as δ in parts per million (ppm) and are relative to tetramethylsilane (TMS) at δ = 0 ppm when using $CDCl_3$ or solvent residual peak (CH_3OH, δ = 3.31 ppm/ DMSO, δ = 2.50 ppm).

Size Exclusion Chromatography.

Size exclusion chromatography (SEC) analysis was performed on an Agilent Infinity II MDS instrument equipped with differential refractive index (DRI), viscometry (VS), dual angle light scatter (LS) and variable wavelength UV detectors. The system was equipped with 2 x PLgel Mixed D columns (300 x 7.5 mm) and a PLgel 5 µm guard column. The mobile phase used was DMF (HPLC grade) containing 5 mM NH_4BF_4 at 50 °C at flow rate of 1.0 ml.min^{-1}. Poly(methyl methacrylate) (PMMA) standards (Agilent EasyVials) were used for calibration between 955,000 – 550 g.moL^{-1}. Analyte samples were filtered through a nylon membrane with 0.22 µm pore size before injection. Number average molecular weights (M_n), weight average molecular weights (M_w) and dispersities ($Đ_M$ = M_w/M_n) were determined by conventional calibration and universal calibration using Agilent GPC/SEC software.

FT-IR Spectroscopy.

The Fourier transform-infrared (FT – IR) analysis was performed on Agilent Cary 630 FTIR spectrometer with diamond ATR system, in the range of 650 to 4000 cm^{-1}.

UV/Visible Spectrophotometer.

UV – Vis spectra were recorded in a Quartz cuvette (volume 700 µl, light path 10 mm x 2 mm, outside dimensions 45 mm x 12.5 mm x 12.5 mm, inside width 2 mm, base thickness 1.25 mm, from Hellma Analytics) using a Cary 60 UV – Vis spectrometer from Agilent at 25 °C, in the range of 200 to 800 cm^{-1}.

Mass Spectrometry.

ESI MS experiments were performed on an Agilent 6130B Single QUAD ESI-LC MS spectrometer in either positive or negative mode with an H2O/MeOH (80:20) eluent, with samples first dissolved in methanol.

1.4. Experimental Methods

Splat Ice Recrystallisation Inhibition Assay.

Splat cooling assays were performed as previously described by Congdon, T *et al.*[1,2] Briefly, a 10 µL sample was dropped 1.40 m onto a chilled glass coverslip, resting on a thin aluminium block placed on dry ice. Upon hitting the coverslip, a wafer with diameter of approximately 10

mm and thickness 10 μm was formed instantaneously. The glass coverslip was transferred onto the Linkam cryostage and held at − 8 °C for 30 minutes. Photographs were obtained using an Olympus CX 41 microscope with a UIS-2 20x/0.45/∞/0-2/FN22 lens and crossed polarisers (Olympus Ltd), equipped with a Canon DSLR 500D digital camera. Images were taken of the initial wafer (to ensure that a polycrystalline sample had been obtained) and again after 30 minutes. Image processing was conducted using ImageJ. The number of ice crystals in the field of view was measured for each photograph. The average (mean) of these three measurements was then calculated to find the mean grain size (MGS) and were reported relative to the MGS of a phosphate buffered saline (PBS) solution pH 7.4.

Photo-reactor.

Photoirradiation experiments were conducted with the EvoluChem™ PhotoRedOx Box device with an internal fan (US Patent #10,906,022) with royal blue 450 – 455 nm and violet 380 nm led light source with Beam Angle 25° and power consumption 18 W.

Freeze/Thaw Cycling Assays.

The indicated PVA (which had been dialysed and freeze dried before) was dissolved in the PBS buffer at the indicated concentration to a volume of 0.5 mL. 10 uL was extracted for the ‘splat assay’ (described above), and the remainder was placed into a standard laboratory freezer (target – 20 °C, but this temperature was not constant for the duration, which was intended to mimic ‘stress’ conditions) for 24 hours. After this time, the vial was removed and allowed to thaw in the laboratory for 15 mins (ambient temperature was 22 °C). A 10 uL sample was again withdrawn, and the ‘splat’ assay ran.

100 Freeze/Thaw Cycling Assays (Figure 8 in main paper).

PVA_{12k} sample was dissolved in PBS buffer at 0.0625 mg mL^{-1} was prepared. 10 uL was extracted for the ‘splat assay’ and the sample froze in a Dewar with liquid nitrogen. After 3 mins the vial was removed and allowed to thaw in the laboratory for 5 mins (ambient temperature was 22 °C). This process was repeated at 10, 40, 60, 80 and 100 freeze/thaw cycles for the same sample.

2. Synthetic Methods

Synthesis of 2-(ethoxycarbonothioyl)sulfanyl propanoate (EXEP).

The RAFT agent was synthesised according to a previously described method.[3] Into a round bottom flask was added ethyl acetate (120 mL) and potassium ethyl xanthate (4.7 g, 0.029 moles, 0.98 eq). After 30 min 2-(methyl bromopropionate) (5.0 g, 0.03 moles, 1 eq) was added dropwise and the solution left to stir for 16 h at 60 °C. The mixture was filtered to remove insoluble KBr and then concentrated *in vacuo*. The crude product was partitioned into DCM (100 ml), washed with water (2x100 mL) and brine solution (1x100 mL) and the organic phase was dried using $MgSO_4$. The solution was filtered and concentrated *in vacuo*, affording the product as a yellow oil. ^{1}H NMR (400 MHz, $CDCl_3$): δ = 1.41 ($C\boldsymbol{H}_3CH_2O$, t, 3H), 1.56 (SCH($C\boldsymbol{H}_3$)CO, d, 3H), 3.75 (COO$C\boldsymbol{H}_3$, s, 3H), 4.40 (S$C\boldsymbol{H}$(CH_3)CO, q, 1H), 4.63 ($CH_3C\boldsymbol{H}_2O$, q, 2H). ^{13}C NMR (400 MHz, $CDCl_3$): δ = 13.66 ($\boldsymbol{C}H_3$CHO), 16.68 (SCH($\boldsymbol{C}H_3$)CO), 47 (S$\boldsymbol{C}$H(CH_3)CO), 52.75 (COO$\boldsymbol{C}H_3$), 70.28 ($CH_3\boldsymbol{C}H_2O$), 171.88 (SCH(CH_3)$\boldsymbol{C}$OO), 211.99 (O$\boldsymbol{C}$(S)S). MS (ESI) m/z: $[C_8H_{14}O_2S_3+Na]^+$ calculated 231.0 g mol^{-1} expected 231.3 g mol^{-1}.

Photo-polymerisation of vinyl acetate using bismuth oxide.

As a representative example, into a 20 mL glass vial were added vinyl acetate (VAc) (2.48 g, 28 mmoL, 300 eq), 2-(ethoxycarbonothioyl)sulfanyl propanoate (EXEP) (0.02 g, 0.096 mmoL, 1 eq), DMSO 2.60 mL and bismuth oxide (Bi_2O_3) (0.013 g, 0.028 mmol, 0.3 eq). The vial was sealed with a subaseal. The sealed vial was incubated at 37 °C with magnetic stirring under 450 − 455 nm using the blue LED source in the photo-reactor for the specified times. An aliquot of crude polymerisation mixture was taken for ^{1}H NMR conversion and $M_{n,NMR}$ analysis in DMSO-*d6*. The sample was diluted in THF (15 mL) and centrifuged 3 times at RCF 22.769xg remove Bi_2O_3 and subsequently precipitated into water to remove the DMSO. The polymer as then precipitated from THF into Hexane (2x50 mL) to remove excess VAc and the polymer dried under high vacuum. Conversion (NMR): 98%, M_n(theoretical): 25300 g moL^{-1}. ^{1}H NMR($CDCl_3$): δ = 1.55 − 1.89 ($C\boldsymbol{H}_2CHOOCH_3$, br, 2H), 1.88 − 2.01 ($CH_2CHOOC\boldsymbol{H}_3$, br, 3H), 4.74 − 4.93 ($CH_2C\boldsymbol{H}OOCH_3$, br, 1H). SEC (THF) M_{nSEC}: 27200 g.mol^{-1} , $Đ_M$ =1.24.

Synthesis of poly(vinyl alcohol).

To obtain the poly(vinyl acetate) (PVA) it is necessary to hydrolyse the PVAc to remove the acetate protecting group. Into a 50 mL round bottom flask equipped with a stir bar, poly(vinyl acetate) (1.00 g) was dissolved in methanol (10 mL) and left to stir until dissolved. Hydrazine

hydrate solution 50 – 60 % (25 mL) was added and the mixture stirred under ambient conditions overnight. Afterwards, the methanol evaporates *in vacuo* and 50 mL milliQ water added in the round bottom flask and the polymer mixture dialysed (MWCO = 1000Da) to remove unreacted hydrazine hydrate and methanol. The purified PVA was then lyophilised to give a white solid as the final product. ^{1}H NMR (400 MHz, DMSO-d_6): δ = 1.21 – 1.56 (**C*H*$_2$**CHOH, br, 2H), 3.75 – 3.94 (CH_2**C*H***OH, br, 1H), (CH_2CH**O*H***, br, 1H), 4.18 – 4.70.

3. Supplementary Characterisation Data

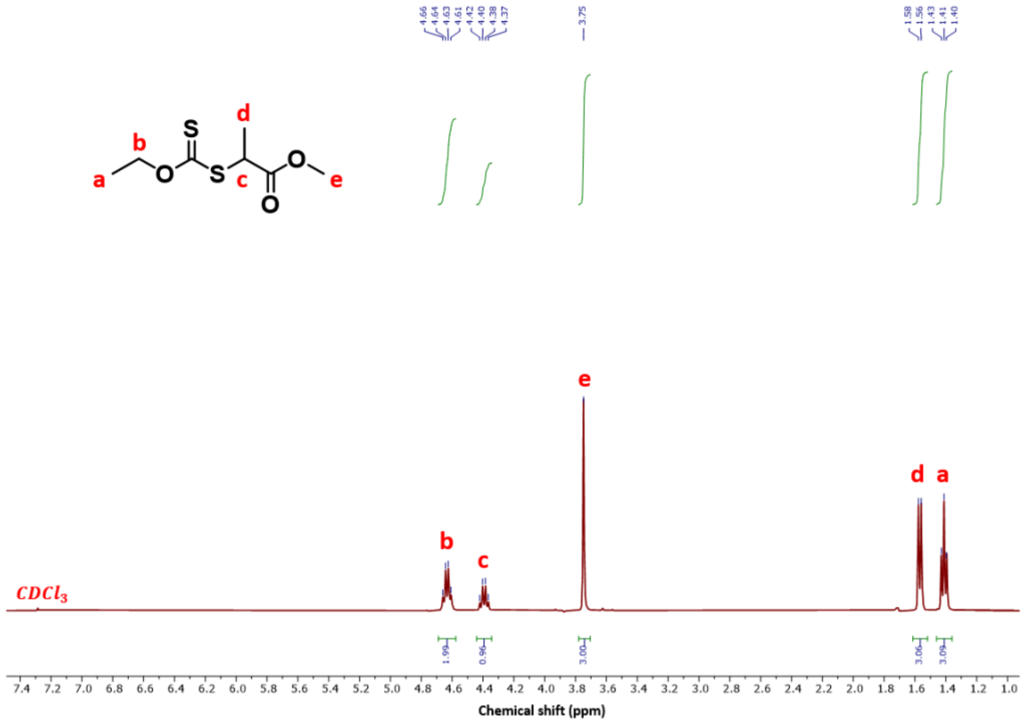

Figure S1. ^{1}H NMR: spectrum of 2-(ethoxycarbonothioyl)sulfanyl propanoate (EXEP) in $CDCl_3$.

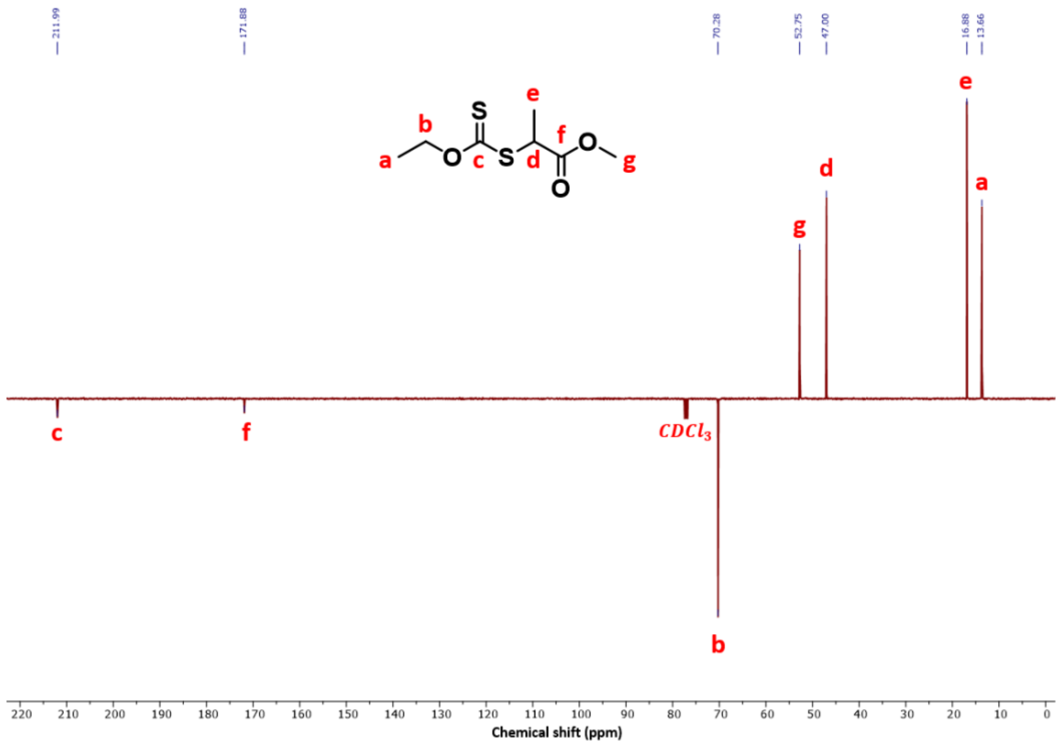

Figure S2. ^{13}C NMR spectrum of 2-(ethoxycarbonothioyl)sulfanyl propanoate (EXEP) in $CDCl_3$.

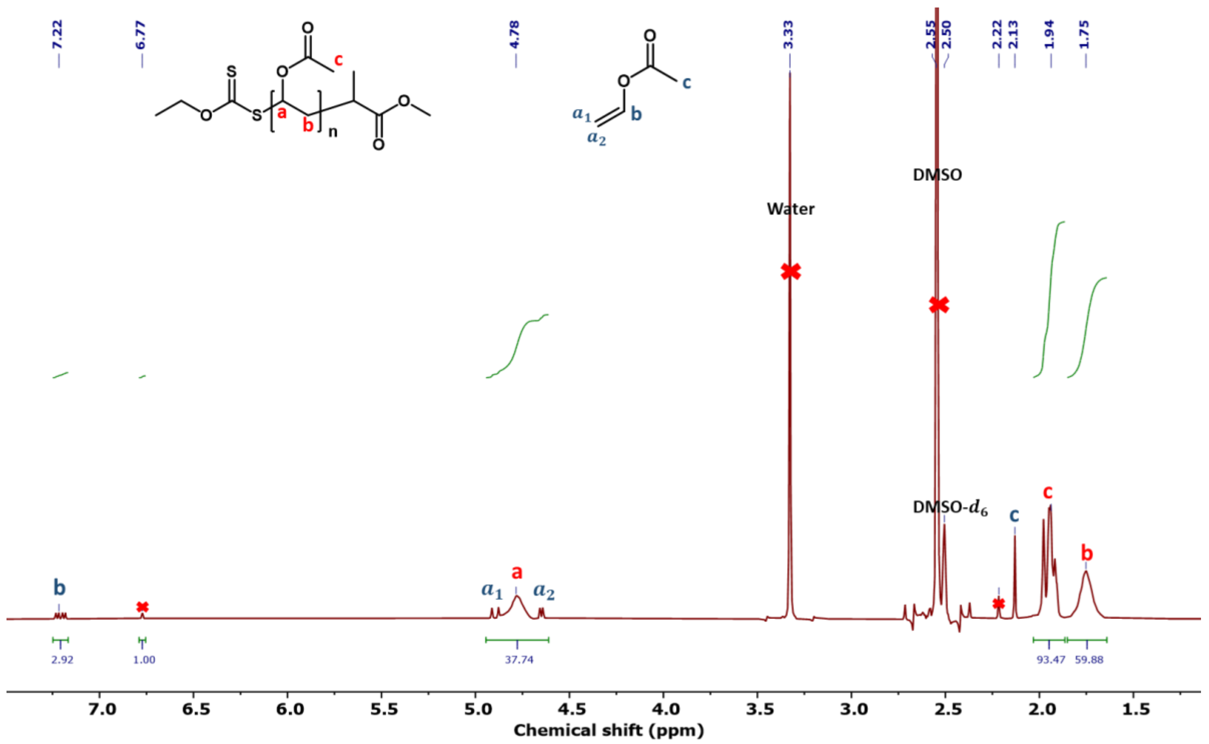

Figure S3. ^{1}H NMR spectrum for $PVAc_{23k}$ showing the VAc monomer conversion using mesitylene standards in DMSO-d_6.

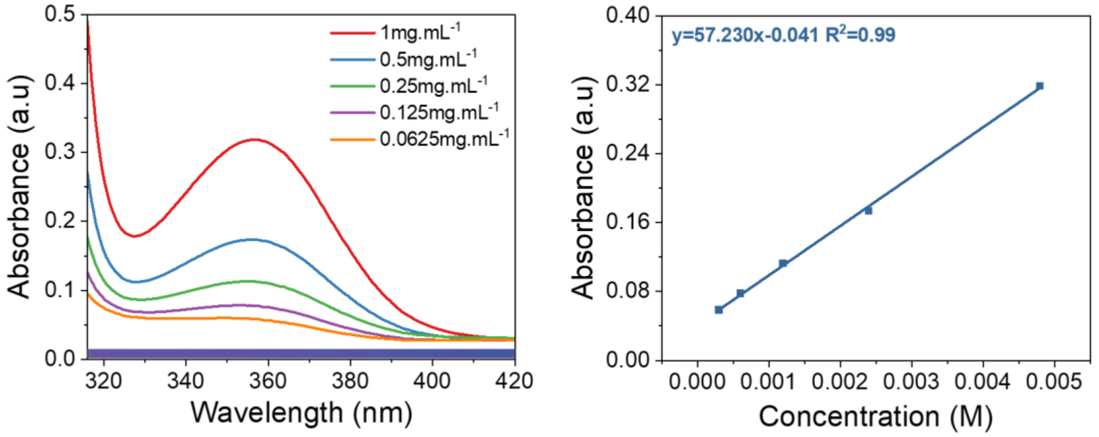

Figure S4. UV − Visible spectra of 2-(ethoxycarbonothioyl)sulfanyl propanoate (EXEP) in DMSO.

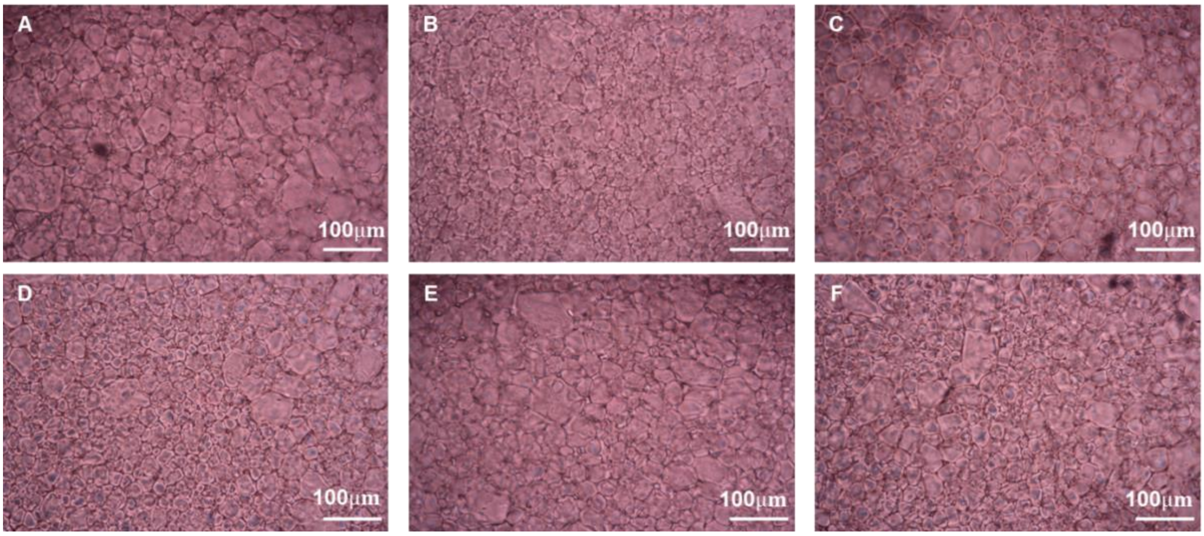

Figure S5. Ice recrystallisation inhibition images of the PVA_{12k} at concentration of 0.0625 mg mL^{-1} after direct dissolution and after multiple freeze/thaw cycles, (A) No freeze/thaw, (B) 20 freeze/thaw cycles, (C) 40 freeze/thaw cycles, (D) 60 freeze/thaw cycles, (E) 80 freeze/thaw cycles, (F) 100 freeze/thaw cycles.

Table S1. PVAc polymers synthesised using the violet (380 nm) light.

PVA	[M]:[CTA]:[Bi_2O_3]	Mn^{SEC} ($g\ mol^{-1}$)	$Đ^{SEC}$	Irradiation time (hours)
**$PVA_{6.3}$**	100:1:0.1	6300	1.29	20

**$PVA_{9.5}$**	150:1:0.15	9500	1.18
**PVA_{16k}**	200:1:0.2	16100	1.29
**PVA_{23k}**	250:0:0.25	23600	1.42

4. References

1. Congdon, T.; Notman, R.; and Gibson, M. I. Antifreeze (glyco) protein mimetic behavior of poly (vinyl alcohol): detailed structure ice recrystallization inhibition activity study. *Biomacromolecules* **2013**, *14* (5), 1578-1586.
2. Biggs, C. I.; Stubbs, C.; Graham, B.; Fayter, A. E.; Hasan, M.; and Gibson, M. I. Mimicking the ice recrystallization activity of biological antifreezes. When is a new polymer "active"?. *Macromolecular bioscience*, **2019**, *19* (7), 1900082.
3. Bell, C. A.; Hedir, G. G.; O'Reilly, R. K.; Dove, A. P. Controlling the Synthesis of Degradable Vinyl Polymers by Xanthate-Mediated Polymerization. *Polym. Chem*. **2015**, *6* (42), 7447–7454.
